# A Biopsychosocial Approach to Understanding Panic Buying: Integrating Neurobiological, Attachment-Based, and Social-Anthropological Perspectives

**DOI:** 10.3389/fpsyt.2021.652353

**Published:** 2021-02-24

**Authors:** Ravi Philip Rajkumar

**Affiliations:** Department of Psychiatry, Jawaharlal Institute of Postgraduate Medical Education and Research, Pondicherry, India

**Keywords:** COVID-19, panic buying, obsessive-compulsive disorder, 5-HTTLPR, hoarding disorder, compulsive shopping, attachment theory

## Abstract

The global COVID-19 pandemic has focused the attention of researchers, civil authority and the general public on the phenomenon of “panic buying,” characterized by the excessive purchase of specific materials—particularly food and hygiene-related products—in anticipation of an expected shortage. This phenomenon has been well-documented in response to several natural and man-made disasters, but its global scope and severity in the context of COVID-19 are unprecedented. This response can negatively impact health, food security, and disease prevention efforts. Attempts to modify such behaviors are more likely to succeed if they are based on insights from both the biomedical and the social sciences. From a biological perspective, the phenomenological overlap between panic buying and psychological disorders such as hoarding disorder and compulsive buying raises the possibility of a shared neurobiological underpinning. Evolutionary models suggest that these behaviors represent an attempt to enhance individual and group survival in the face of a threatened scarcity of resources. These phenomena may be influenced by specific genetic variants which are also implicated in hoarding-related psychological disorders. From a psychological perspective, attachment theory provides a conceptual framework that serves as a bridge between prior life adversity, current deprivation, and an increased attachment to material objects. Such a framework is of relevance when considering panic buying during the COVID-19 pandemic, which has been associated with significant disruptions in attachment bonds. From a social-anthropological perspective, hoarding and related behaviors have been associated with social exclusion and rejection, as well a lack of social support. These risk factors have affected large sections of the general population in the context of the COVID-19 pandemic and the governmental responses to it. This perspective also emphasizes the symbolic significance of the hoarded objects themselves. In this paper, an attempt is made to integrate these three perspectives and thereby formulate a biopsychosocial model of panic buying in response to this global health crisis. The existing scientific literature on panic buying is examined in the light of this model. Finally, suggestions are proposed as to how this model might inform social strategies aimed at preventing or reducing panic buying.

## Introduction

### The Phenomenon of “Panic Buying” During the Global COVID-19 Pandemic

For the past 1 year, the world has faced a global outbreak of acute respiratory illness of unprecedented extent and impact. This illness, caused by the novel betacoronavirus SARS-CoV-2, has been designated COVID-19. Clinical manifestations of COVID-19 range from asymptomatic viral carriage to severe respiratory illness, with the latter outcome being more common in the elderly and those with significant medical comorbidities (1). At the time of writing this paper (January 12, 2021), over 86 million cases of COVID-19, and over 1.8 million deaths due to this disease, have been reported globally (2).

In an effort to contain the spread of this disease, local and federal authorities worldwide have resorted to large-scale containment measures such as lockdowns, “stay-at-home orders,” and restrictions on commercial, educational, religious, and other public activities. Though deemed necessary by governments and experts, these measures have often led to widespread socioeconomic disruption, difficulties in accessing healthcare, and shortages of food and other essential supplies (3).

Against this background, a significant proportion of the general population has experienced various forms of psychological distress, such as symptoms of anxiety, depression, and post-traumatic stress. The frequency, severity and correlates of these phenomena have already been documented extensively in several systematic reviews (4–6). Besides these well-recognized phenomena, reports of “panic buying” in response to the COVID-19 pandemic have been reported in the literature since March 2020, with the earliest published reports coming from Asian countries (7–9). Wang et al. (9). Panic buying, also referred to as “stockpiling,” is characterized by the purchase of “unusually large amounts of products,” in anticipation of or during a natural or man-made disaster, related to a fear of shortage of unavailability of the concerned products, which are usually food or hygiene-related items (10, 11). Panic buying has been documented as a local response to phenomena such disease outbreaks or typhoons (12, 13), but has occurred on an unprecedented scale in the context of COVID-19 and the attendant restrictions imposed in an attempt to contain the spread of the disease (14).

Various explanatory models have been advanced to account for this phenomenon (11, 14). Before examining these, a brief review of the existing literature on the scope of this phenomenon, and the factors associated with it, is in order.

### Frequency and Correlates of Panic Buying

Changes in purchasing behavior during the COVID-19 pandemic were commonly reported in the general population; in a study of Spanish consumers, over 60% reported such changes. However, only a small proportion of them exhibited actual panic buying (15). A review of media reports (14) found descriptions of panic buying from over 20 different geographical regions, encompassing both developing and developed countries. This paper found a predominance of reports (97.6%) from countries with a high level of urbanization and industrialization, though this may simply reflect a reporting bias. A study from Portugal reported stockpiling in 36% of respondents (16). In a report from the United States, the most common stockpiled materials were toilet paper (63%), canned foods (59%), staple foods such as rice and bread (53–57%), bottled water (57%), and medications (53%) (17).

A number of factors have been associated with an increased risk of panic buying. These may be conveniently classified as follows:

- *Individual factors*: male sex (18); increased extraversion and neuroticism; low conscientiousness and openness (19); need to belong (20); need for safety or reassurance (21–23); anxiety or worry (8, 21, 23); reduced adherence to social distancing (17); conservative attitudes (17);
- *Social and economic factors*: local severity of the pandemic (13, 20); perception of scarcity or of an increase in price (21); lack of trust in public authorities (19, 21); misinformation (20, 21); restrictions on internal movement (13); pre-existing psychiatric illness (24).

### Proposed Explanations for Panic Buying and Their Limitations

Alongside the descriptive research on panic buying, summarized above, several authors have speculated on the possible causes or mechanisms underlying this complex behavior. From the perspective of survival psychology, acquiring essential supplies during an actual or threatened disaster is an adaptive behavior; however, when this behavior is influenced by excessive anxiety or fear of the “unknown,” or of “losing control” over the situation, the result is irrational decision-making and panic buying (8, 11, 21). Alchin (25) has suggested that excessive exposure to displays of “panic buying” by others, either directly or through the media, can lead to the activation of evolutionarily primitive brain pathways which suppress higher-level “pro-social” behaviors, leading to a behavior is socially inappropriate. His model has also highlighted the potential role of social learning or imitation in reinforcing maladaptive cognitions regarding the risk of food shortage or scarcity of other essential supplies. From a broader socioeconomic perspective, Keane and Neal (13) have highlighted the role of local and international patterns of viral transmission, as well as government-imposed restrictions on internal (but not external) movement, in influencing panic buying.

While these explanations have a great deal of merit to them, they are deficient in three aspects. First, they are—to a certain extent—unduly reductive, and fail to consider the way in which biological, individual and social factors may interact. As a result, suggestions to improve panic buying that are derived from one model, such as excessive anxiety, may not be effective in areas where social factors are of greater significance. Second, these models fail to account for the significant variability in panic buying that has been reported across individuals, regions and countries. Third, these models do not explain the specific phenomenon of panic buying, which is a unique and discrete behavior pattern—at best, they provide general explanations for psychological distress and maladaptive coping during the COVID-19 pandemic.

In order to address these limitations, an outline for a more comprehensive model was proposed by Arafat et al. (21). In this model, the precipitating event (a disaster, such as a pandemic or earthquake) interacts with psychological processes (appraisal and processing of the event) and social factors (such as cultural and political variables) to produce a sense of threat (resource scarcity) leading to panic buying. However, they have noted that this model needs to be verified by further research.

This paper takes the position that the broad outline proposed by these authors is essentially correct. However, it may be possible to refine it further and define areas for research, prevention and intervention by taking into account recent advances in the biological and social sciences. These advances and their implications will be discussed in section Divergent Theoretical Perspectives on Panic Buying below, and an attempt to integrate them will be presented in section Summary: Integrated Biopsychosocial Model of Panic Buying.

## Divergent Theoretical Perspectives on Panic Buying

### Neurobiology

From a biological perspective, Alchin (25) has attempted to explain panic buying in terms of the polyvagal theory proposed by Porges (26). According to this theory, perception of threat by the brain generates “primitive” fearful responses through activation of the autonomic nervous system and hypothalamic-pituitary-adrenal axis; however, if a situation is subsequently determined to be safe, this response can be overridden through a putative “social engagement system” (SES), mediated through the vagus nerve, which dampens stress responses and facilitates prosocial behaviors. However, exposure to continuous threatening stimuli—especially through the media, including social media—may overwhelm the SES, leading to maladaptive behaviors that are socially harmful. This theory, though plausible and backed by advances in neuroscience, functions better as a general theory of maladaptive behavior during the COVID-19 pandemic, and does not explain why a specific form of behavior (panic buying) should arise.

A more fruitful approach may be obtained by examining the similarities between panic buying and certain known psychological disorders—more specifically, obsessive-compulsive disorder (OCD) and related conditions such as hoarding disorder and compulsive buying. These latter conditions are considered to lie on the “OCD spectrum” and share genetic and neurobiological links with it (27–30). Hoarding disorder, whether considered as a discrete entity or as a subset of OCD, is characterized by the irrational accumulation of materials, usually of a non-essential nature, to an extent that causes functional impairment (31). Though the symptoms of these disorders are recognized as irrational and excessive by sufferers, they can also be understood from an evolutionary perspective as a dysfunctional variant of adaptive behaviors related to threat detection and harm avoidance (32). More specifically, hoarding behavior has been conceptualized as a form of altruistic behavior that helps in maintaining a supply of scare resources, particularly in the face of a large-scale disaster or other cause of scarcity (33, 34). The argument, in brief, runs as follows: individuals with an innate tendency to hoard essential materials during a crisis would ensure a survival advantage for both individuals and their community as a whole, particularly in more “primitive” or traditional societies. On the basis of analogies with animal behavior, a similar hypothesis has been proposed by Miguel and Ligabue-Brown (35); their proposal requires no putative link to OCD.

From this perspective, it is possible to understand the emergence of stockpiling behavior in the face of actual or anticipated disasters, and especially in the context of a global crisis such as COVID-19. However, this explanation is missing a crucial factor: what causes this behavior to cross the “threshold of rationality” and take on an excessive form, namely panic buying? A number of neurobiological factors may underlie this phenomenon, including a paradoxical increase in prefrontal gray matter (36) and over-activation of specific brain regions, such as the anterior cingulate and dorsolateral prefrontal cortices (37). A similar model implicating under-activity of the medial prefrontal cortex and overactivity of nucleus accumbens has been postulated for compulsive buying (38). These changes may be reflected in higher-order constructs such as difficulties in emotion regulation, and intolerance of uncertainty or distress. From this viewpoint, the numerous uncertainties and negative emotions associated with the COVID-19 pandemic and its attendant socioeconomic changes may trigger aberrant neural activity in susceptible individuals, leading to an aberrant form (panic buying) of an otherwise useful behavior (purchasing essential materials) (39).

In addition, regional or cross-national variations in panic buying may be explicable in terms of genetic variations influencing brain structure and function, which account for 36% of the variance in hoarding disorder (40); further, there is a correlation of over 40% between genetic vulnerability for hoarding disorder and OCD (41). In this context, it is worth noting a consistent association between the long (*l*) allele of the serotonin transporter functional polymorphism 5-HTTLPR, located on chromosome 17, and vulnerability to OCD in certain subgroups (42). This genetic variant is of particular significance to behavioral responses to the COVID-19 pandemic, as there is some evidence that it was subject to selection pressures caused by infectious disease, and that it may influence behavioral responses to the threat of infection (43). These aspects will be discussed further in section Summary: Integrated Biopsychosocial Model of Panic Buying.

It can be argued that though phenomenologically similar, panic buying may not necessarily exist on a continuum with OCD; to date, no literature has reported an association between the two, or an increased risk of panic buying in OCD patients. This could be explained by the alternate hypothesis that hoarding may have different evolutionary roots from OCD in general (35). In the model proposed by these authors, human genes homologous to those involved in hoarding behavior in birds may be activated via epigenetic modification during traumatic or threatening situations. This would trigger a tendency to accumulate possessions and derive a sense of safety from them. Another neurobiological hypothesis arises from the similarity of panic buying to compulsive buying. In situations where opportunities for natural rewards from the environment are lacking (due to containment measures or social distancing), repeated shopping or buying might activate the mesolimbic dopaminergic pathway, leading to a perception of reward and reinforcing this behavior.

### Attachment Theory

If hoarding behavior has remote evolutionary origins, and can be triggered by a low tolerance to (or an inability to self-regulate) psychological distress, what are the specific psychological mechanisms or pathways linking distress and panic buying? In examining this association in the context of COVID-19, a useful perspective can be obtained from the field of attachment theory, as formulated by John Bowlby (44, 45). According to attachment theory, many psychological disorders have their origins in disruptions of attachment bonds in early childhood, and can be triggered or exacerbated by the disruption of attachment bonds later in life (46). Contemporary neuroscience has identified a certain degree of overlap between the brain regions involved in attachment behavior and those implicated in hoarding disorder, such as the anterior cingulate cortex (37, 47). Further, there is evidence that parental abuse, neglect or separation in early childhood, which are all associated with significant disruption of attachment bonds, are associated with an undue emotional attachment to possessions, which in turn is linked to the severity of subsequent hoarding behavior (48). Similarly, later experiences of social exclusion (49), trauma in interpersonal relationships (50), or poor social support (51) have been associated with the development and maintenance of hoarding behavior in adult life. This may be mediated through insecure adult attachment, leading to negative affective states which in turn trigger an increased attachment to, and desire to accumulate, certain possessions (51, 52). Similar phenomena have also been reported in compulsive buyers, though they are less well-characterized (53, 54).

The addition of these facts to the neurobiological framework discussed in section Neurobiology allows a more complete picture to emerge. In a given individual, genetic vulnerability interacts with disruption in early attachment bonds to influence the structure and function of discrete brain regions implicated in hoarding disorder, which is manifested in higher-order psychological constructs such as difficulties in emotion regulation, distress tolerance and tolerance of uncertainty, or a heightened attachment to possessions. However, the emergence and persistence of hoarding symptoms requires further disruptions or deficits in interpersonal attachment in later life (adolescence or adulthood). This is particularly relevant to the COVID-19 pandemic, where infection control measures have led to the disruption of existing interpersonal bonds on a global scale (3, 45). In such contexts, even individuals who had never previously exhibited hoarding behavior may develop an increased attachment to, and urge to accumulate, certain possessions—leading, not to hoarding disorder *per se*, but to panic buying. In this context, it is worth noting that many of the individual psychological variables identified as being linked to panic buying, such as high neuroticism and low openness and conscientiousness (17), have been specifically associated with disturbances in attachment both in children and in young adults (55).

Though this model is both evidence-based and plausible, it remains deficient in one significant aspect. Hoarding disorder is characterized by the accumulation of materials which are non-essential, while panic buying is characterized by the accumulation of objects which are actually or potentially essential; though excessive, it is not pathological because lacks the irrationality which is characteristic of hoarding disorder; neither is it unrelated to external threats, as in compulsive buying disorder. In order to explain this discrepancy, it is now necessary to turn to social-anthropological perspectives on these phenomena.

### Social and Anthropological Perspectives

**Figure 1:**
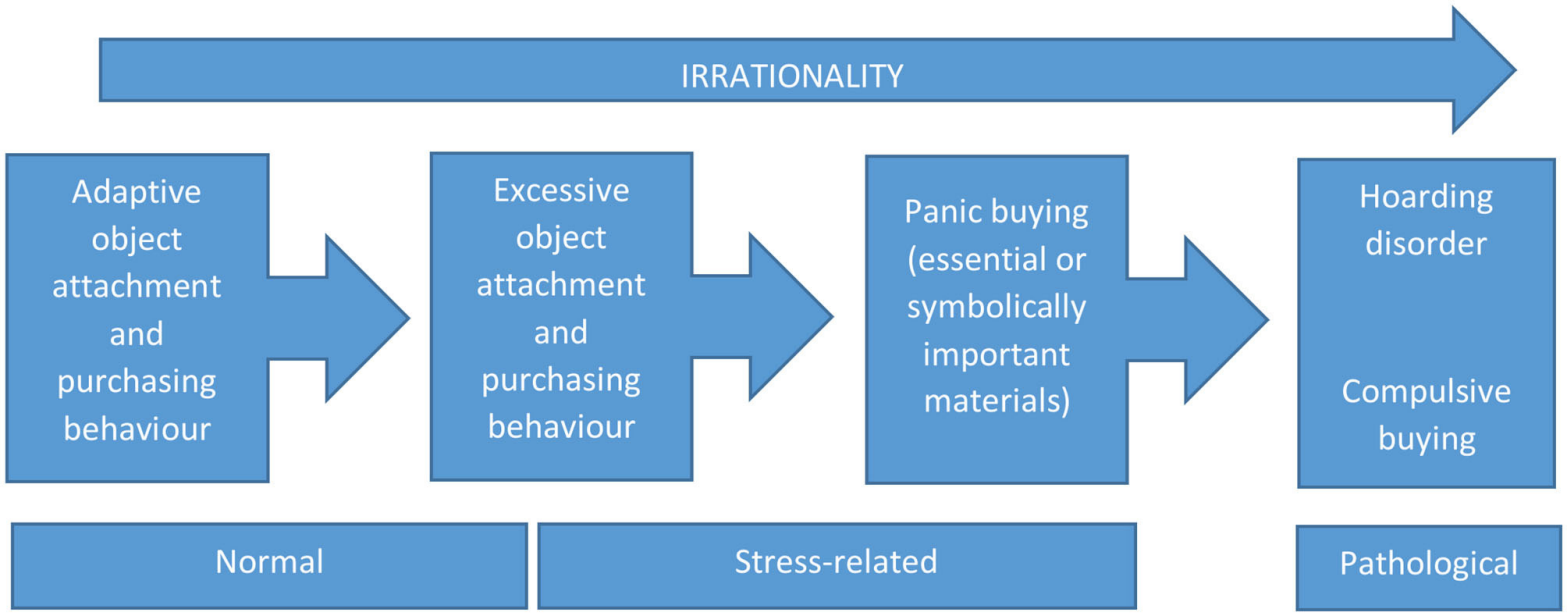
A proposed continuum between normal object attachment and purchasing behavior, excessive forms of such behavior, panic buying, and psychiatric disorders such as hoarding and compulsive buying.

Hoarding disorder is characterized by a pathological form of attachment to material objects; however, object attachment varies significantly across individuals and cultures, and is influenced by a variety of factors. These include exposure to specific life events, individual and cultural beliefs, physical health, and cognitive functioning. From an anthropological perspective, an object that appears “non-essential” or even “worthless” may have a symbolic significance for a given individual (56, 57). In other words, it may be unduly reductive to create a dichotomy between “essential” and “non-essential” forms of object accumulation. Instead, these behaviors may exist on a spectrum or continuum, ranging from normal object attachment, to rational stockpiling of essential supplies in the face of threat, to excessive stockpiling of essential supplies in the same context, to overt panic buying, and finally to hoarding disorder or compulsive buying where the element of rationality is almost completely absent (16, 53, 57) (see Figure 1). In support of this notion, it is useful to note that the materials hoarded in panic buying may vary significantly in perceived “usefulness” across cultures. For example, the hoarding of guns and ammunition, which have symbolic significance in terms of safety and self-defense, may occur in certain Western countries (17) but not in Asian countries (21). Moreover, the value attached to possessions in general varies across cultures. Western countries generally assign a higher level of importance to material possessions, which are often portrayed as linked to success, happiness and popularity (58); compulsive shopping has been reported more frequently in such cultures (59), as has panic buying (14, 21).

These theories of symbolic meaning must be placed in the broader social framework of the COVID-19 pandemic, which has been characterized not only by large-scale disruptions of social bonds (as outlined in section Attachment Theory) but by economic disruptions leading to unemployment and poverty (60, 61). In turn, these disruptions have been associated with increased rates of specific social problems, such as domestic violence (62) and social exclusion or stigmatization (63) at the individual level. The large-scale social unrest and hardship occasioned by this pandemic is thus “translated” into a number of individual-level stressors, most of which have been associated with the initiation or maintenance of hoarding behavior (49–51). At a symbolic level, a lack of trust in local or federal authorities—which may be worsened by the disruption of social bonds (45)—can further increase the likelihood of panic buying, as can a social learning effect based on imitation (25). The interactions between these factors are likely to be complex and non-linear.

With these details in place, it is now possible to formulate a more comprehensive model.

## Summary: Integrated Biopsychosocial Model of Panic Buying

**Figure 2:**
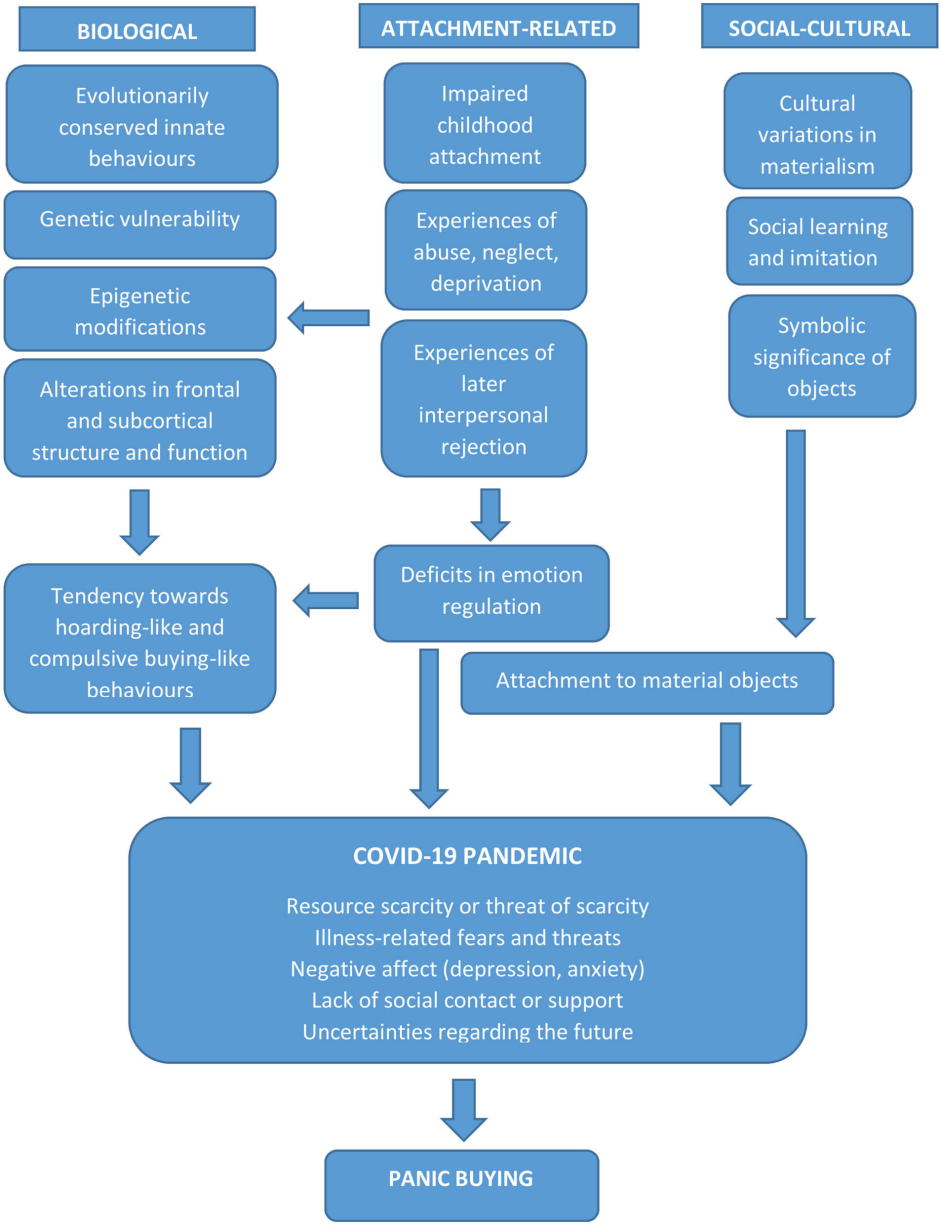
A proposed biopsychosocial model of panic buying, showing the interaction of biological, attachment-related and social-cultural factors with the proximate stresses and trauma associated with the COVID-19 pandemic.

In brief, what is being proposed in this paper can be outlined in the following three propositions (Figure 2):

- At the most basic level, stockpiling behavior has distant evolutionary roots, and probably evolved as a response to actual or threatened scarcity of resources in primitive or traditional societies. This behavior exists on a continuum with more pathological forms (obsessive-compulsive disorder, hoarding disorder) and is at least partly determined by genetic and epigenetic factors. The neural circuitry underlying this behavior—both in its adaptive and maladaptive forms—is complex, and involves higher-order brain regions such as the dorsolateral prefrontal and anterior cingulate cortices. This “instinctive” response is activated by the perception of a threatened or actual scarcity, and has been reported on a greater or lesser scale in a variety of disaster-related contexts.
- At the individual, higher-order level, stockpiling behavior—and its pathological variants, hoarding and compulsive buying—also serve secondary psychological functions such as the regulation of negative emotions, low self-esteem, or distress related to uncertainty. Individual susceptibility to these behaviors is influenced by both genetic factors and early childhood adversity involving the disruption of attachment bonds. In adult life, the actual or threatened disruption of attachment bonds through social isolation, exclusion, disharmony, aggression or a general lack of support is an important trigger—and maintaining factor—for these behaviors.
- At the social level, the large-scale social and economic disruptions caused by the COVID-19 pandemic and governmental responses to it lead to an increase in actual or threatened disruptions of family and community bonds. This leads to stockpiling, and in extreme cases, panic buying, the severity of which is related to individual biological and psychological diatheses. Additional social factors influencing this behavior include social learning through imitation, and a lack of trust in the ability of civil authorities to ensure an adequate supply of essential products. Cultural factors may both influence the likelihood of panic buying and the nature of the materials purchased, some of which may have symbolic significance.

Though the exact extent of the relative contributions of biological, psychological and social factors remains an open question, the framework outlined above provides a useful guide to further research on this phenomenon. It is also worth nothing that some factors may operate at multiple levels. For example, at an individual level, the serotonin transporter polymorphism 5-HTTLPR influences the susceptibility to obsessive-compulsive and related behaviors. However, at a broader level, it also appears to influence cultural patterns, with the *l* allele predominating in more individualistic societies and the *s* allele in societies characterized by a higher degree of collectivism; further, these cross-national variations may themselves have arisen as a result of survival advantages conferred during outbreaks of infectious disease (43).

Further, it should be emphasized that though the model presented here draws on research into certain psychiatric disorders, panic buying itself is neither a psychiatric disorder nor a symptom of one. Rather, it is an excessive form of a “normal” adaptive behavior (63) that may lie on a continuum with certain disorders, just as sadness and grief exist on a continuum with depression (64). As a majority of the research on panic buying has arisen in the context of this pandemic, it is possible that the proposal outlined here may require correction in whole or in part in the light of further evidence (65). It is also important to note that panic buying does not occur in a vaccum. The attitudes, emotional responses and behaviors of merchants and civil authorities during a pandemic need to be studied in order to obtain a more complete picture of how these factors influence, and are influenced by, the behavior of “panic buyers” (66).

## Research Findings Explained By The Integrated Model

Though research explicitly focused on the model presented here does not exist yet, there are several findings from the existing literature that lend support to it:

- Several studies have reported either a fresh onset of OCD symptoms (67) or an increase in pre-existing OCD symptoms (68, 69) during the COVID-19 pandemic. This is predicted by the current model which views panic buying and OCD-related behaviors (hoarding and buying) on a continuum. The countries represented in these reports are those (such as Canada, the United States, and European countries) which have also reported increases in panic buying (8, 14).
- The onset of OCD symptoms during the COVID-19 pandemic has been associated with an intolerance of uncertainty and distress (70) as well as with an increase in and a higher level of perceived threat (71, 72) have both been associated with the emergence of *de novo* OCD symptoms during the pandemic. These psychological mechanisms are related to both hoarding behavior (73) and panic buying (21), which would be expected if these behaviors have common psychological roots.
- The majority of media reports of panic buying have come from countries where the *l* allele of the serotonin transporter polymorphism 5-HTTLPR is predominant (21, 43). This genetic variant has been associated with OCD and related conditions (41, 42).
- A need for reassurance has been identified as one of the key variables influencing panic buying (14, 24); this variable is also a key mediator of the link between negative mood states and the emergence of OCD symptoms (74).
- Perceived scarcity of resources and lack of access to them have both been linked with panic buying (13, 21, 66). This finding is consistent both with the psychological and social aspects of this model, in which the accumulation of objects symbolizes safety and security, as well as with the postulated evolutionary roots of hoarding and related behaviors (32, 35).
- A qualitative study of reports from retailers dealing with “panic buyers” during the initial phase of the pandemic found many commonalities with the factors highlighted in this model: exaggerated threat perception, intolerance of uncertainty, a need for safety, disrupted social bonds, social learning, and an actual or symbolic value assigned to the objects purchased (66).
- An in-depth study of individuals indulging in impulsive buying outside the context of the COVID-19 pandemic outlined the specific psychological and social factors reported as important by these individuals: a need to control negative mood states, the perceived actual or symbolic value of the objects purchased, and a perceived lack of availability of the purchased objects (75). These factors were similar to those reported by individuals indulging in panic buying during the pandemic (21, 24).

## Avenues For Future Research and Practical Implications

If this model is wholly or partly correct, it has important implications for strategies aimed at minimizing or preventing it. There are a number of avenues for research that would serve to either confirm or refute the model proposed here, either entirely or partly. At an individual level, the occurrence of panic buying in patients with OCD, hoarding disorder and compulsive buying could be examined. Similarly, the occurrence of panic buying at a higher rate in the relatives of individuals with these conditions would support a possible link between them. Genetic and brain imaging studies could identify potential overlapping factors between panic buying and these disorders, though these should be conducted in strict adherence to ethical guidelines. Studies of childhood and adult attachment in individuals exhibiting panic buying, in relation to a control group, could clarify the role of interpersonal and object attachment. Finally, multi-national studies could identify the role of cultural and symbolic factors in influencing both the occurrence of panic buying and the nature of the objects that are preferentially purchased.

When considering the real-world applications of this model, it is worth noting that many of the risk factors for panic buying identified by researchers can be accommodated within this framework. Individual factors such as neuroticism, need for reassurance and a high level of anxiety are—as would be predicted by the literature on hoarding disorder—associated with higher levels of panic buying (19, 21, 23, 24), as are local conditions characterized by higher levels of social isolation or interpersonal stress (13, 21). The model proposed here is also entirely consistent with the frameworks outlined by Alchin (25) and Arafat et al. (21) and is in many senses complementary to them; while Alchin's model highlights the role of threat perception and social learning in panic buying, and Arafat et al.'s model integrates psychological and social factors, the current model provides further insights regarding possible biological, psychological and social processes that underpin these constructs.

Though the biological and early childhood factors identified in this paper may not be directly amenable to intervention during the COVID-19 pandemic, the psychological and social factors identified as triggering or maintaining panic buying provide fruitful avenues for strategies aimed at preventing or minimizing this behavior (76, 77). These may be briefly enumerated as follows:

- Concerted efforts must be made by civil authorities—both at the local and federal level—to mitigate the economic hardships caused by the COVID-19 pandemic. This may take the form of direct financial assistance, strategies aimed at ensuring a reliable and equitable supply of essentials to communities, providing alternative forms of temporary employment, and the like. The exact nature of these interventions may vary according to local economic and cultural conditions.
- Given the key role played by anxiety and intolerance of uncertainty in influencing these behaviors, accurate information on the pandemic and the measures necessary to contain it must be disseminated in a form that is understandable and culturally appropriate (76). Myths and misconceptions which may lead to stigmatization, exclusion or social avoidance should be corrected. The preparation of educational materials should be done in collaboration with experts in the fields of public health, infectious disease, and health psychology. These materials should also frame adherence to safety measures as an altruistic or even heroic act, to ensure a “positive” form of social learning (77).
- When infection control measures are necessary, these should be explained in advance, and should not be enforced in an arbitrary or unduly punitive manner, to avoid undermining public trust. Instead of punishments for “offenders,” positive incentives for adherence to hygienic measures or social distancing may be offered.
- As pre-existing psychological and social vulnerabilities may exacerbate the impact of the pandemic and trigger these behaviors, the above two interventions should be provided more urgently in areas already characterized by high rates of economic deprivation, unemployment, or social unrest.
- Given the hypothesized inverse relationship between social support and panic buying, efforts must be made to ensure at least a certain degree of social contact between individuals, while respecting basic safety precautions. Particular attention must be paid to institutions with a particular social or cultural significance, such as schools, colleges and places of worship. A balance should be struck between reasonable safety measures and continued access to these, given their direct and symbolic importance to large numbers of individuals.
- The media should avoid undue sensationalism and speculation when reporting on the pandemic, and should ensure the accuracy of all published information to the farthest extent. As is done for other social problems such as suicide, they should provide information on avenues for help or assistance and not merely highlight problems or present them as insoluble (76).
- Continued access to healthcare, especially for those with pre-existing psychiatric disorders on the anxiety or obsessive-compulsive spectrum, should be ensured. Though telemedicine-based models may be useful in this regard, they are not always feasible in certain settings, and direct consultations may need to be offered, while ensuring adherence to hygienic measures.
- Healthcare workers interacting with individuals who indulge in panic buying, or who are communicating with the public on this matter, should try to understand the above perspectives. When doing so, they should attempt to provide clear information and reassurance; to link distressed individuals with available physical and psychological resources; and to explain this behavior as an understandable but excessive response to a crisis rather than taking a judgmental or medicalizing stance. They must also attempt to teach anxiety reduction or stress management techniques where applicable, as these might regulate the putative psychological and epigenetic factors related to panic buying.
- Some of these suggestions are in line with the existing recommendations of experts from various countries (76, 77). These medically and socially oriented proposals should ideally be implemented alongside more logistic, supply chain-related solutions. The latter have already been implemented in several countries (78), though their efficacy requires further evaluation.

## Conclusions

Though it may be viewed as an extreme response to an extreme situation, the phenomenon of panic buying shines a light on the evolutionary roots of long-standing, conserved patterns of behavior, their primary and secondary functions, and their sensitivity to individual and social stressors. Panic buying is in itself not a pathological condition but an excessive form of an adaptive behavior (79, 80). However, it may share common roots with certain psychiatric disorders. It may be possible to prevent or minimize panic buying through social strategies informed by an integrated bio-psycho-social model, as has been outlined in this paper. As the model presented here is of a theoretical nature, it should be interpreted and applied with prudence until it is subjected to more rigorous empirical testing. If this does occur, is hoped that the insights presented here would aid both the scientific and the larger human community when confronted with future disease outbreaks or other disasters.

## Author Contributions

RR developed the concept for this review, carried out the literature search, wrote the paper, and proofread it. This paper represents the author's original work and has not been submitted for publication elsewhere.

## Conflict of Interest

The author declares that the research was conducted in the absence of any commercial or financial relationships that could be construed as a potential conflict of interest.
